# Alterations of dendritic cell subsets in the peripheral circulation of patients with cervical carcinoma

**DOI:** 10.1186/1756-9966-29-78

**Published:** 2010-06-18

**Authors:** Feng Ye, Yan Yu, Yuting Hu, Weiguo Lu, Xing Xie

**Affiliations:** 1 Women's Reproductive Health Key Laboratory of Zhejiang Province, Women's Hospital, School of Medicine, Zhejiang University, Xueshi Rd#2, Hangzhou, 310006, China; 2 Women's Reproductive Health Key Laboratory of Zhejiang Province; Department of Gynecologic Oncology, Women's Hospital, School of Medicine, Zhejiang University, Xueshi Rd#2, Hangzhou, 310006, China

## Abstract

Patients with cervical carcinoma (CC) are frequently immunocompromised. Dendritic cells (DCs) are potent antigen-presenting cells. Using multicolor flow cytometry, the percentages of CD11c+ (DC1) and CD123+ (DC2) subsets, were determined in the peripheral blood of 37 patients with cervical carcinoma (CC), 54 patients with CIN, and 62 healthy individuals. A substantial reduction of circulating dendritic cells and accordingly immunodepression may be associated with increased IL-6 and TGF-β in serum. These findings could give expression to the immunosuppression of circulating dendritic cells in patients with CC and CIN, thus, may indicate novel aspects of cervical carcinoma immune evasion.

## Introduction

Cervical carcinoma (CC) is the second most common cancer among women worldwide. Approximately 371,200 new cases are diagnosed each year, and nearly 200,000 deaths are attributable to the disease [1-4]. Cervical carcinoma and its precursor lesions, cervical intraepithelial neoplasia (CIN), are virus-related neoplasms. As such, their initiation and promotion is associated with persistent infection by oncogenic human papillomavirus (HPV) [5,6]. Although early stage cervical carcinoma can be cured by radical surgery or radiotherapy with similar effectiveness [7], up to 35% of patients will develop advanced metastatic disease [8] for which treatment results are poor. Immunotherapeutic agents may provide a novel therapeutic strategy for the treatment of recurrent and metastatic disease. Cervical carcinoma patients obviously fail to mount an efficient cytotoxic T cell response against HPV antigens. This is probably due to low expression levels of both viral protein and MHC molecules [9,10] as well as to lack of costimulatory molecules crucial for naive T cell priming by the tumor cells [11]. For these reasons, current research aims to develop more efficient immunotherapy to stimulate an antitumor immune response. In this context, one approach toward developing an effective immunotherapeutic regime for cervical carcinoma may be through the manipulation of antigen-presenting cells, such as dendritic cells (DCs). DCs are potent, professional antigen-presenting cells which can initiate a primary immune response to antigens by naive T cells [12]. Several lines of evidence suggest that DCs loaded with various tumor antigens, such as tumor fragments or antigen peptides, or with antigen genes by way of retrovirus or adenovirus vectors, are capable of activation and expansion of tumor-specific T cells in vitro [5,13-15]. To date, only a few clinical trials of DC vaccination have been reported in cancer patients, with disappointing results. In addition to the immunodeficiency of the patients, several other limitations are currently hindering the potential of this technique, including attaining pure DCs, loading the DCs with tumor antigen, and transducing the DCs with tumor genes [5,14,16-18].

DCs, as the most potent antigen presenting cells, play a central role in the initiation and regulation of immune responses, Which are detected using multicolor flow cytometry, electron microscope or immunocytochemistryImmunocytochemistry Immunocytochemistry Immunocytochemistry and so on. However, human DCs are not a homogenous population. Besides inducing anti-tumor immunity, DCs can induce tumor-special anergy or tolerance [18-21]. DCs originate from CD34+ hematopoietic stem cells (HSC). Myeloid dendritic cells (DC1) and plasmacytoid DCs (DC2) are the two principal subpopulations of human DCs, and their characteristics vary greatly in phenotype, migration, and function. DC1 cells are effective T cell stimulators, inducing a tumor specific immune response; however, the function of DC2 cells is uncertain. They not only stimulate tumor specific immune responses, they also contribute to tumor immune tolerance. It has been suggested that CD11c+DC1 cells primarily induce Th1 differentiation, whereas DC2 cells, which express the receptor for IL-3 (CD123), mainly promote a Th2 response. Many studies indicate that in a tumor microenvironment, DCs both decrease in quantity and are impaired in function. Both DC populations were significantly lower in patients with cancer than in healthy donors [22-25]. DC subsets may be used differentially in immune responses to various antigens, including tumor-associated antigens. However, little is known about the frequency or function of these two subsets of DCs in cervical carcinoma patients.

Tumors lack specific antigens and can hide or change their antigens to escape immune surveillance. They can also manipulate dendritic cell subset distributions and subvert tumor immunity by secreting inhibitory cytokines such as IL-2, IL-4, IL-10, IL-6, TFG-β, VEGF, and IFN-γ. Some of these are produced by human tumor cells themselves, whereas others are not only produced by tumor cells but also induced systemically by tumor cell-derived products. TGFβ acts as a stimulator of tumor invasion by promoting extracellular matrix production and angiogenesis, stimulating tumor proliferation, and inhibiting host immune functions [26]. IL-6 has an immunosuppressive role in cancer patients by inhibiting the development of DCs [27]. VEGF is a strong mitogen for endothelial cells and raises vascular permeability; it takes part in the neovascularization of the tumor tissue [28]. Elevated IL-10 concentrations in serum contribute to an impaired antitumor immune response [29]. These cytokines may directly or indirectly affect the function of DCs.

In the current study, we want to know the change of subsets of DCs in CC and the health. And we hope to get the message from the trend. we investigated the proportions of these two DC subsets in the peripheral circulation of 37 patients with cervical carcinoma, 54 patients with CIN, and 62 healthy individuals using multicolor flow cytometry. We detected the expression of CD123, CD11c, HLA-DR, CD80 and CD86 on the surface of DCs. We also investigated the levels of the cytokines IL-10, IL-6, TFG-β, and VEGF in serum to examine the claim that the low proportion and impaired maturity of freshly isolated dendritic cell subsets from patients with cervical cancer correlates with increased levels of cytokines in their serum.

## Materials and methods

### Patients

All patients were from the Women's Hospital School of Medicine at Zhejiang University (Hangzhou, China) with histologically confirmed primary cervical carcinoma and were recruited between June 2006 and May 2007. All the patients have no prior therapy restrictions including surgery chemiotherapy and radiotherapy. All the patients have no other complications, so their vital sign and basic lab tests are normal. The stages of all CIN patients seletected are CINI-III. The stages of all CC patients seletected are early stage(Ia1 to Ib2). Controls were randomly selected from healthy women seen for gynecologic examinations at the Women's Hospital School of Medicine at Zhejiang University during the period when women with cervical cancer and CIN were enrolled. Control selection criteria included no positive findings during the gynecological examination, no history of cancer, age matching to the patients and residence in Zhejiang Province. A total of 90 patients were studied, 37 with cervical carcinoma and 58 with CIN including 54 CINII-III and 4 CINI. For too few CINI, they were not being statistics. All women included in the study provided written informed consent.

### Flow Cytometry Analysis

2 ml peripheral blood (PB) were taken into heparinized tubes (sodium heparin). Peripheral blood mononuclear cells (PBMCs) were isolated by density gradient centrifugation on Lymphoprep (Amersham Bioscience, Sweden) for 25 min at 600 g at room temperature. PBMCs were collected and washed twice in phosphate-buffered saline. The cells were stained using the following antibodies: anti-CD11c-FITC, anti-CD123-PE, anti-HLA-DR-PE-Cy5, anti-CD80-FITC, and anti-CD86-PE. Respective IgG isotype controls were run for each specimen. Isolated cells (5 × 10^5^) were incubated for 30 min at 4°C with monoclonal antibodies specified against surface antigens and washed twice in PBS containing 0.2 mm ethylenediaminetetraacetic acid (EDTA) and 0.5% bovine serum albumin (BSA). Freshly isolated peripheral blood mononuclear cells were analyzed by flow cytometry for quantitation and immunophenotyping of dendritic cell subsets. The data were analyzed using Cell Quest software (Becton Dickinson, San Jose, California, USA). The myeloid DCs (DC1) were identified as a population of mononuclear cells expressing CD11c+, but without expression of CD123. Lymphoid DCs (DC2) were identified as CD123+, but without expression of CD11c.

### ELISA

Sera from 37 patients with cervical cancer, 54 patients with CINII-III and 62 controls were collected for cytokine quantitation. Concentrations of serum IL-6, IL-10, VEGF and TGF-β were measured by ELISA according to the manufacturerâ€™s instruction (BD Biosciences, San Diego, CA). The assay sensitivities for IL-6, IL-10, VEGF and TGF-β are 2 pg/ml, 19 pg/ml, 5 pg/ml and 15.6 pg/ml. All assays were conducted in duplicate.

### Statistical Analysis

Statistical analysis was performed by ANOVA with Bonferroni modification. Differences were considered significant at p values < 0.05.

## Results

### Dendritic cell subsets in patients and controls

**Figure 1:**
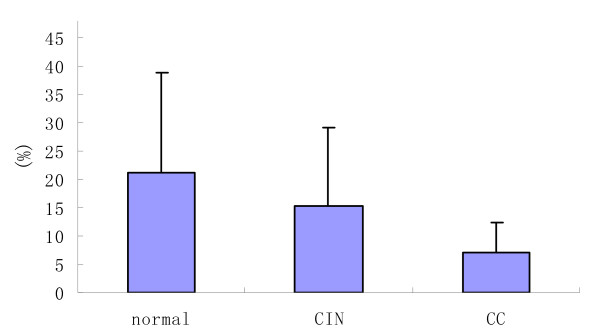
**The percentage of DC1 in patients with CC, CIN and controls**.

**Figure 2:**
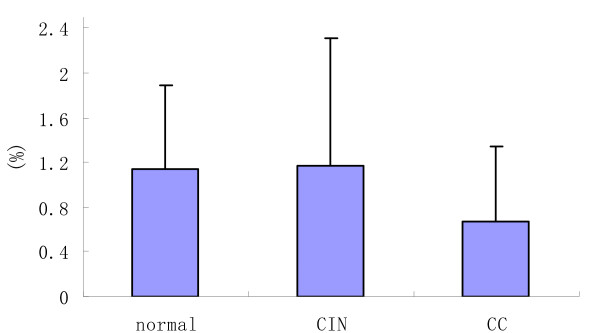
**The percentage of DC2 in patients with CC, CIN and controls**.

In this study we detected both myeloid (CD11c+) and lymphoid (CD123+) cells in peripheral blood of women with cervical carcinoma or CINII-III and in controls. The proportions of dendritic cell subsets are given in Table 1 and Figure 1, Figure 2. In patients with cervical carcinoma, DC1 constituted 7.00 ± 5.49% of total PB mononuclear cells; in CINII-III they were 15.38 ± 13.63%, and in controls they were 21.22 ± 17.69%. The percentage of DC1 was significantly lower (P < 0.05) in patients with cervical carcinoma than in the CIN and control groups. There were no significant differences (P > 0.05) in the percentage of DC1 between the CIN groups and the controls.

**Table 1:** The percentage of DC1 and DC2 in patients with CC, CINII-III and controls

	Normal (n = 62)	CINII-III (n = 54)	CC (n = 37)	P		
CD11c+(DC1)	21.22 ± 17.69	15.38 ± 13.63	7.00 ± 5.49	0.096*	0.000**	0.000***
CD123+(DC2)	1.14 ± 0.75	1.17 ± 1.14	0.67 ± 0.484	0.392*	0.012**	0.087***

*Normal vs CINII~III; ** Normal vs CC; *** CINII~III vs CC

P of the three groups: CD11c+(DC1): P = 0.000, F = 16.839; CD123+(DC2): P = 0.042, F = 3.248

In patients with cervical carcinoma, DC2 constituted 0.67 ± 0.484% of total PB mononuclear cells; in women with CINI-III they were 1.17 ± 1.14%, and in controls they were 1.14 ± 0.75%. The percentage of DC2 was significantly lower (P < 0.05) in patients with cervical carcinoma than in the control group. The percentage of DC2 was not significantly different (P > 0.05) between patients with cervical carcinoma and the CIN group. There were also no significant differences (P > 0.05) in the percentage of DC2 between the CIN groups and the controls. We found that the proportions of DC2 were lower in patients with cervical carcinoma in comparison with the CIN and the controls; the CIN and the controls were almost equivalent.

### The variation of surface markers in DCs of patients with CC, CIN and controls

**Figure 3:**
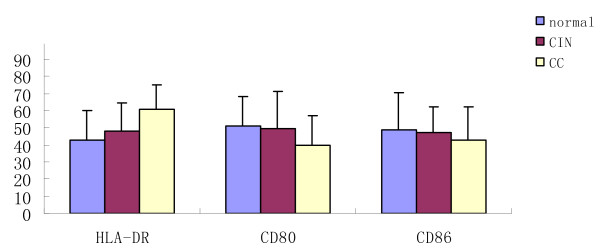
**The functional immunophenotypings of DCs in patients with CC, CIN and controls**.

To further characterize DCs in cancer patients, we next determined their expressions of the surface markers HLA-DR, CD80, and CD86 by flow cytometry. The expressions of these antigens are shown in Table 2 and Figure 3. We found the HLA-DR expression in the CIN group (48.09 ± 16.07%) was higher than that in the healthy individuals (42.70 ± 17.53%) and highest in patients with cervical carcinoma (60.59 ± 14.64%). It was significantly higher (P < 0.05) in the CC group compared to the CIN group and the controls. But no significant differences (P > 0.05) between the CIN groups and the controls were observed.

**Table 2:** The functional immunophenotypings of DCs in patients with CC, CINII-III and controls

	Normal (n = 62)	CINII-III (n = 54)	CC (n = 37)	P		
HLA-DR	42.70 ± 17.53	48.09 ± 16.07	60.59 ± 14.64	0.082*	0.000**	0.001***
CD80	51.2 3 ± 17.16	49.52 ± 21.74	39.59 ± 17.39	0.633*	0.004**	0.017***
CD86	49.02 ± 21.58	46.92 ± 15.24	42.54 ± 19.51	0.803*	0.157**	0.111***

*Normal vs CINII~III; ** Normal vs CC; *** CINII~III vs CC

P of the three groups: HLA-DR: P = 0.000, F = 13.634; CD80: P = 0.012, F = 4.587;

CD86: P = 0.241, F = 1.438

We also detected the expression of CD80 and CD86 on the surface of DCs. The expression of CD80 and CD86 in the CIN group (CD80: 49.52 ± 21.74%; CD86: 46.92 ± 15.24%) was lower than that of the healthy individuals (CD80: 51.23 ± 17.16%; CD86: 49.02 ± 21.58%), and lowest in patients with cervical carcinoma (CD80: 39.59 ± 17.39%; CD86: 42.54 ± 19.51%). There was significantly lower (P < 0.05) CD80 expression in the CC groups than in the controls, and also significantly lower expression (P < 0.05) in the CC group than in the CIN group. But no significant differences (P > 0.05) between the CIN groups and the controls were observed. There were no significant differences in CD86 between any groups. DCs from the peripheral blood of cancer patients thus exhibit decreased expression of these costimulatory molecules as compared to controls.

### Cytokine secretion in CC, CIN and controls

**Figure 4:**
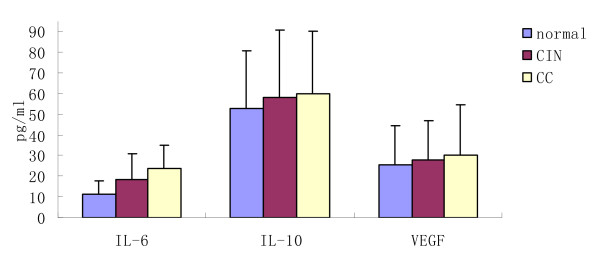
**The functional immunophenotypings of DCs in patients with CC, CIN and controls**.

**Figure 5:**
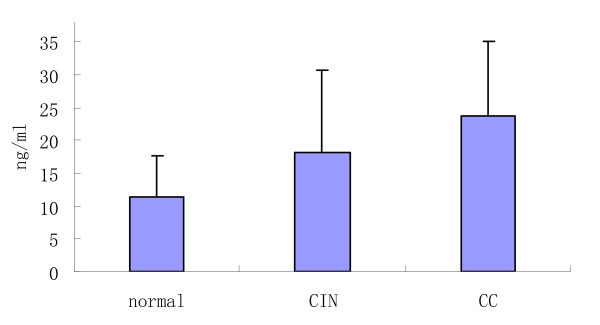
**The serum TGFβ secretion in patients with CC, CIN and controls**.

We next investigated cytokine secretion in patients with CC and CIN compared to controls. The levels of these cytokines are shown in Table 3and Figure 4, Figure 5. Women with CIN (18.19 ± 12.58 pg/mL) had significantly higher IL-6 levels in their peripheral blood than did controls (11.29 ± 6.36 pg/mL); IL-6 levels were highest in women with CC (23.67 ± 11.36 pg/mL). There were significant differences between any two groups.

**Table 3:** The serum cytokines secretion in patients with CC, CINII-III and controls

	Normal (n = 62)	CINII-III (n = 54)	CC (n = 37)	P		
IL-6 ( pg/ml)	11.29 ± 6.36	18.19 ± 12.58	23.67 ± 11.36	0.000*	0.000**	0.013***
TGFβ ( ng/ml )	5.60 ± 4.83	6.41 ± 5.20	18.22 ± 12.18	0.598*	0.000**	0.000***
IL-10 ( pg/ml )	52.69 ± 28.27	57.95 ± 32.94	60.18 ± 29.92	0.358*	0.243**	0.735***
VEGF ( pg/ml )	25.54 ± 19.13	27.92 ± 19.13	30.39 ± 24.19	0.365*	0.436**	0.976***

*Normal vs CINII~III; ** Normal vs CC; *** CINII~III vs CC

P of the three groups:IL-6: P = 0.000, F = 17.712; TGFβ: P = 0.000, F = 21.671;

IL-10: P = 0.450, F = 0.802; VEGF: P = 0.601, F = 0.511

Similar observations were found for TGF-β. The level of TGF-β in the CIN group (6.41 ± 5.20 pg/mL) was higher in comparison to the healthy individuals (5.60 ± 4.83 pg/mL) and highest in patients with cervical carcinoma (18.22 ± 12.18 pg/mL). It was significantly higher (P < 0.05) between the CC groups and the controls. It was also significantly higher (P < 0.05) between the CC groups and the CIN group. But no significant differences (P > 0.05) between the CIN groups and the controls were observed.

No obvious variation was observed in levels of IL-10 and VEGF. The levels of IL-10 and VEGF in the CIN group (IL-10: 57.95 ± 32.94 pg/mL; VEGF: 27.92 ± 19.13 pg/mL) were higher in comparison to the healthy individuals (IL-10: 52.69 ± 28.27 pg/mL; VEGF: 25.54 ± 19.13 pg/mL) and highest in patients with cervical carcinoma (IL-10: 60.18 ± 29.92 pg/mL; VEGF: 30.39 ± 24.19 pg/mL). There were no significant differences between any two groups.

Patients with CC and CIN thus have higher levels of these suppressive cytokines than the controls.

## Discussion

The ability of tumor cells to evade host immune system control can be ascribed to many mechanisms, including deletion of tumor-specific cytotoxic T-lymphocytes and recruitment of regulatory T-lymphocytes and inhibitory cell types. In addition, cancer patients may present a defect in the host immune system [4,30,31]. One of the targets of this defect is represented by professional APC; an impaired DC function in cancer patients has been reported by several groups [32-34]. Tumors achieve this suppressive effect on DC by secreting tumor-derived factors, as recently described [27,29,35]. Human DCs are phenotypically and functionally heterogeneous. The ability to identify and enumerate DCs and their subsets in tumor tissue and in the peripheral circulation of patients with cancer appears to be fundamental for the understanding of the role of these cells in the host antitumor responses.

Firstly, we showed that patients with cervical carcinoma and CIN exhibit a significant decrease in the absolute number of circulating DCs when compared to healthy controls. The reduction affects both of the two main subsets of DCs circulating in the PB. The most striking observation of the current study was a relative decrease in the percentage of CD11c+DC cells (DC1) in the peripheral circulation of CC patients. The percentage of DC1 was significantly lower (P < 0.05) in patients with cervical carcinoma than in the CIN and control groups. This is in agreement with other studies [23,24,36].

Secondly, we also found that the proportions of CD123+DC cells (DC2) were lower in patients with cervical carcinoma in comparison with the CIN and the controls; the CIN and the controls were almost equivalent, and there was not significantly different (P > 0.05) between the CC and the CIN. It is seemed that DC2 do not decrease noticeably in the CIN, although they were decreased in the CC like DC1.

As the classic antitumor cells, DC1 were induced to apoptosis by tumor if there was no tumor intervention or enhancement of DC1. The loss of DC1 thus correlates with tumor burden. DC2 possessing both antitumor and immunosuppression displayed a little differently in process of tumor. The side of immunosuppression may permit or promote the development of tumor [33,34].

Our findings indicate that, in cervical carcinoma patients, decreased numbers of these cells closely correlate with disease stage and tumor progression. The decrease in circulating DCs may have functional consequences on the production of cytokines and on antigen presentation to naive T-cells. The reason for the decreased frequency of these two subsets of DCs in patients with CC remains unknown. It may be that tumors induce apoptosis in DCs by direct contact, or tumors may inhibit the differentiation of DCs in vivo by secreting soluble factors.

Several studies have demonstrated that DCs in tumor patients are not able to induce primary T-cell responses. Antigen-specific Treg cells were over-represented at tumor sites and mediated antigen-specific, local, immune suppression, thus inhibiting the function of anti-cancer T effector cells [37,38]. We showed that the DCs upregulated their MHC class II molecules (HLA-DR) in response to tumor-associated antigens, although DCs from patients with CC and CIN exhibit more upgraded HLA-DR than controls. However, the level is moderated, which is different from other studies(29,36). Lee et al. found that in women with human papillomavirus (HPV)-related cervical squamous intraepithelial lesions (SILs) or atypical squamous cells of undetermined significance (ASCUS), peripheral blood DC1 and monocyte-derived dendritic cells (MDDCs), but not DC2 cells, expressed low levels of HLA-DR [39]. In our study, there is no significant difference in HLA-DR between the CIN groups and the controls, but in the CC group, the expression of HLA-DR increased. HPV DNA is found in 90% of all cervical cancers. DC2 can be activated by this virus, which causes them to undergo maturation. This process enhances their antigen presentation potential by upregulating MHC class II molecules. However, even in fully mature DC2 cells, levels of MHC II and costimulatory molecules remain significantly lower than in DC1 cells [40]. This may be the reason that the expression of HLA-DR increased and the level is moderated. In addition, all circulating dendritic cell subsets exhibited low CD80 and CD86 expression, which is concordant with other reports [29,41]. Our data confirm that in cancer patients, DCs display an 'immature' phenotype with lower levels of accessory signals for T cell activation such as CD80 and CD86 than those expressed on DCs from normal healthy controls. This may reduce their ability to stimulate T cells. The antitumor effect of DCs is dependent on their level of activation and maturation. Lack of costimulatory molecules in the presence of TCR occupancy leads to T cell tolerance.

Several studies have demonstrated the effects of individual tumor-derived or tumor-induced cytokines on DC function as they relate to the immune response to malignant tumors [42]. In our study, higher levels of all cytokines under investigation, especially TGFβ and IL-6, were detected in patientsâ€™ sera compared to controls. This is inversely correlated with circulating DC1 and DC2, indicating a possible effect of these cytokines on DCs. TGFβ and IL-6 are closely related to the invasion and metastasis of cancer. They thus might play pivotal but opposing roles in the host tumor interaction that, together with other immunomodulating components, determines the outcome for the development of local tumor immunity [43]. Many studies in vitro indicate that these tumor-derived regulatory cytokines have been shown to inhibit DC development and to impair DC function[27,29,41,44]. DCs generated in vitro from progenitors purified from cancer patients are capable of stimulating T-cell responses, but blood DCs isolated from the same patients are deficient in their APC capacity[27,45]. Our study indicates that the defect in circulating DC from cervical carcinoma could, at least in part, be the result of decreased frequency of competent DC and the accumulation of immature cells with poor APC function. Tumors may also inhibit circulating DCs by secreting immunosuppressive cytokines.

In summary, we showed that the two subsets of DCs in PB of patients with cervical carcinoma are significantly reduced, and that this decrease correlates with an increase in tumor-derived regulatory cytokines. The findings reported here are relevant due to the large effort devoted to harnessing blood DCs for the immunotherapy of cancer. Our data should also be taken into account when assessing immune competence, as it suggests that it might not be appropriate to use the peripheral blood DC compartment as a source of cells for DC-based cancer immunotherapy protocols.

## Competing interests

The authors declare that they have no competing interests.

## Authors' contributions

FY carried out cells separated by the multicolor flow cytometry and drafted the manuscript. YY carried out the blood collection from patients and health. YH participated in the ELISA assays. WL participated in the design of the study and performed the statistical analysis. XX conceived of the study, and participated in its design and coordination. All authors read and approved the final manuscript
